# 3-(Adamantan-1-yl)-4-phenyl-1-[(4-phenyl­piperazin-1-yl)meth­yl]-1*H*-1,2,4-triazole-5(4*H*)-thione

**DOI:** 10.1107/S1600536811055711

**Published:** 2012-01-11

**Authors:** Ebtehal S. Al-Abdullah, Hanadi H. Asiri, Ali El-Emam, Seik Weng Ng

**Affiliations:** a Department of Pharmaceutical Chemistry, College of Pharmacy, King Saud University, Riyadh 11451, Saudi Arabia; b Department of Chemistry, University of Malaya, 50603 Kuala Lumpur, Malaysia; c Chemistry Department, Faculty of Science, King Abdulaziz University, PO Box 80203 Jeddah, Saudi Arabia

## Abstract

The title mol­ecule, C_29_H_35_N_5_S, displays a chair-shaped piperazine ring, as well as an approximately planar triazole ring (r.m.s. deviation = 0.001 Å) whose phenyl substituent is nearly perpendicular to the mean plane of the five-membered ring [dihedral angle = 88.9 (1)°]. The substituents on the piperazine ring occupy equatorial sites. In the crystal, the adamantyl cage is disordered over two sets of sites with a major component of 67.8 (5)%. Weak inter­molecular C—H⋯S hydrogen bonding is present in the crystal.

## Related literature

For the synthesis and applications of 3-(1-adamant­yl)-4-substituted-5-mercapto-1,2,4-triazole derivatives, see: El-Emam & Ibrahim (1991 ▶).
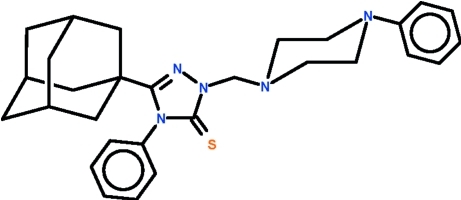


## Experimental

### 

#### Crystal data

C_29_H_35_N_5_S
*M* *_r_* = 485.68
Monoclinic, 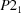

*a* = 11.3342 (5) Å
*b* = 8.4744 (3) Å
*c* = 13.7868 (6) Å
β = 103.864 (4)°
*V* = 1285.65 (9) Å^3^
*Z* = 2
Mo *K*α radiation
μ = 0.15 mm^−1^
*T* = 100 K
0.40 × 0.35 × 0.30 mm

#### Data collection

Agilent SuperNova Dual diffractometer with an Atlas detector
Absorption correction: multi-scan (*CrysAlis PRO*; Agilent, 2010 ▶) *T*_min_ = 0.941, *T*_max_ = 0.956
8952 measured reflections
5539 independent reflections
5155 reflections with *I* > 2σ(*I*)
*R*_int_ = 0.022

#### Refinement

*R*[*F* ^2^ > 2σ(*F* ^2^)] = 0.060
*wR*(*F* ^2^) = 0.154
*S* = 1.02
5537 reflections
398 parameters
169 restraints
H-atom parameters constrained
Δρ_max_ = 0.72 e Å^−3^
Δρ_min_ = −0.56 e Å^−3^
Absolute structure: Flack (1983 ▶), 2382 Friedel pairs
Flack parameter: 0.01 (11)

### 

Data collection: *CrysAlis PRO* (Agilent, 2010 ▶); cell refinement: *CrysAlis PRO*; data reduction: *CrysAlis PRO*; program(s) used to solve structure: *SHELXS97* (Sheldrick, 2008 ▶); program(s) used to refine structure: *SHELXL97* (Sheldrick, 2008 ▶); molecular graphics: *X-SEED* (Barbour, 2001 ▶); software used to prepare material for publication: *publCIF* (Westrip, 2010 ▶).

## Supplementary Material

Crystal structure: contains datablock(s) global, I. DOI: 10.1107/S1600536811055711/xu5420sup1.cif

Structure factors: contains datablock(s) I. DOI: 10.1107/S1600536811055711/xu5420Isup2.hkl

Supplementary material file. DOI: 10.1107/S1600536811055711/xu5420Isup3.cml

Additional supplementary materials:  crystallographic information; 3D view; checkCIF report

## Figures and Tables

**Table 1:** Hydrogen-bond geometry (Å, °)

*D*—H⋯*A*	*D*—H	H⋯*A*	*D*⋯*A*	*D*—H⋯*A*
C19—H19*B*⋯S1^i^	0.99	2.84	3.593 (4)	133

Symmetry code: (i) 
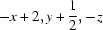
.

**Fig. 1.**
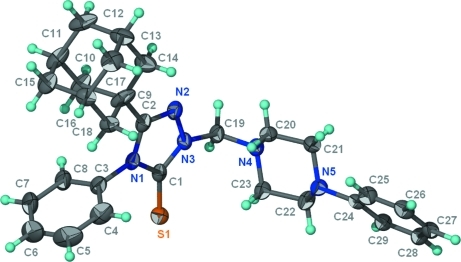
Thermal ellipsoid plot (Barbour, 2001) of C29H35N5S at the 70% probability level; hydrogen atoms are drawn as spheres of arbitrary radius. The minor disorder component is not shown.

## supplementary crystallographic information

### Comment

We reported the synthesis, anti-inflammatory and analgesic properties of 3-(1-adamantyl)-4-substituted-5-mercapto-1,2,4-triazole derivatives (El-Emam & Ibrahim, 1991). The triazole ring, which possesses a secondary nitrogen site next to a double-bond sulfur, is capable of undergoing a Mannich reaction with an *N*-substituted piperazine derivative to yield a new class of chemotherapeutic compounds. The C_29_H_35_N_5_S molecule (Scheme I, Fig. 1) displays a chair-shaped piperazine ring, as well as a planar triazole ring whose phenyl substituent is nearly perpendicular to the mean plane of the five-membered ring (dihedral angle 88.9 (1) °). The substituents on the piperazine ring occupy equatorial sites. The adamantyl cage is disordered.

### Experimental

5-(1-Adamantyl)-4-phenyl-1,2,4-triazole-3-thiol was synthesized according to a reported procedure (El-Emam & Ibrahim, 1991). The compound (2 mmol), 1-phenylpiperazine (2 mmol) and a 37% formaldehyde solution (0.5 ml) in ethanol (8 ml), was heated for 15 minutes. Stirring was continued for 12 h at room temperature. The product was filtered, washed with water, dried, and recrystallized from ethanol to yield 816 mg (80%) of the title compound as colorless crystals, m.p. 598–500 K.

### Refinement

Carbon-bound H-atoms were placed in calculated positions [C–H 0.95 to 1.00 Å, *U*_iso_(H) 1.2*U*_eq_(C)] and were included in the refinement in the riding model approximation.

The adamantyl cage is disordered over two positions; 1,2- and 1,3-C–C distances were tightly restrained to 1.500±0.002 and 2.450±0.005 Å; their temperature factors were refined. The disorder refined to a major component of 67.8 (5)%. The anisotropic temperature factors were restrained, with those of the minor component atoms being more tightly restrained than those of the major component atoms.

Omitted owing to bad disagreement were (-5 - 2 8), (-7 - 2 9),(-6 - 3 6) (1 - 8 0) and (-5 - 3 6).

The Flack parameter was calculated from 2382 Friedel pairs.

### Crystal data

**Table:**

C_29_H_35_N_5_S	*F*(000) = 520
*M**_r_* = 485.68	*D*_x_ = 1.255 Mg m^−^^3^
Monoclinic, *P*2_1_	Mo *K*α radiation, λ = 0.71073 Å
Hall symbol: P 2yb	Cell parameters from 5784 reflections
*a* = 11.3342 (5) Å	θ = 2.4–27.5°
*b* = 8.4744 (3) Å	µ = 0.15 mm^−^^1^
*c* = 13.7868 (6) Å	*T* = 100 K
β = 103.864 (4)°	Wedge, colorless
*V* = 1285.65 (9) Å^3^	0.40 × 0.35 × 0.30 mm
*Z* = 2	

### Data collection

**Table:**

Agilent SuperNova Dual diffractometer with an Atlas detector	5539 independent reflections
Radiation source: SuperNova (Mo) X-ray Source	5155 reflections with *I* > 2σ(*I*)
Mirror	*R*_int_ = 0.022
Detector resolution: 10.4041 pixels mm^-1^	θ_max_ = 27.6°, θ_min_ = 2.8°
ω scan	*h* = −14→14
Absorption correction: multi-scan (CrysAlis PRO; Agilent, 2010)	*k* = −10→11
*T*_min_ = 0.941, *T*_max_ = 0.956	*l* = −13→17
8952 measured reflections	

### Refinement

**Table:**

Refinement on *F*^2^	Secondary atom site location: difference Fourier map
Least-squares matrix: full	Hydrogen site location: inferred from neighbouring sites
*R*[*F*^2^ > 2σ(*F*^2^)] = 0.060	H-atom parameters constrained
*wR*(*F*^2^) = 0.154	*w* = 1/[σ^2^(*F*_o_^2^) + (0.0713*P*)^2^ + 1.5877*P*] where *P* = (*F*_o_^2^ + 2*F*_c_^2^)/3
*S* = 1.02	(Δ/σ)_max_ = 0.001
5537 reflections	Δρ_max_ = 0.72 e Å^−^^3^
398 parameters	Δρ_min_ = −0.56 e Å^−^^3^
169 restraints	Absolute structure: Flack (1983), 2382 Friedel pairs
Primary atom site location: structure-invariant direct methods	Flack parameter: 0.01 (11)

### Fractional atomic coordinates and isotropic or equivalent isotropic displacement parameters (Å^2^)

**Table:**

	*x*	*y*	*z*	*U*_iso_*/*U*_eq_	Occ. (<1)
S1	0.82856 (7)	0.50082 (16)	0.02316 (5)	0.02700 (18)	
N1	0.8127 (2)	0.7494 (3)	0.14650 (19)	0.0224 (5)	
N2	1.0027 (2)	0.8295 (4)	0.1988 (2)	0.0281 (6)	
N3	0.9950 (2)	0.7016 (3)	0.1347 (2)	0.0253 (6)	
N4	1.1767 (2)	0.5280 (4)	0.1622 (2)	0.0254 (6)	
N5	1.2418 (2)	0.2718 (4)	0.29827 (19)	0.0244 (6)	
C1	0.8801 (3)	0.6498 (4)	0.1015 (2)	0.0224 (6)	
C2	0.8918 (3)	0.8563 (4)	0.2051 (2)	0.0246 (6)	
C3	0.6821 (3)	0.7415 (4)	0.1239 (3)	0.0297 (7)	
C4	0.6262 (4)	0.6461 (5)	0.1808 (3)	0.0438 (10)	
H4	0.6729	0.5857	0.2345	0.053*	
C5	0.4988 (4)	0.6406 (6)	0.1573 (5)	0.0613 (15)	
H5	0.4586	0.5758	0.1956	0.074*	
C6	0.4320 (4)	0.7274 (6)	0.0803 (5)	0.0637 (15)	
H6	0.3458	0.7228	0.0653	0.076*	
C7	0.4887 (4)	0.8215 (6)	0.0240 (5)	0.0593 (14)	
H7	0.4416	0.8820	−0.0294	0.071*	
C8	0.6154 (3)	0.8283 (5)	0.0452 (3)	0.0404 (9)	
H8	0.6550	0.8920	0.0059	0.049*	
C9	0.8586 (2)	0.9902 (3)	0.2700 (2)	0.0366 (8)	
C10	0.7951 (4)	1.1115 (4)	0.1942 (2)	0.0408 (14)	0.678 (5)
H10A	0.8508	1.1462	0.1530	0.049*	0.678 (5)
H10B	0.7228	1.0630	0.1493	0.049*	0.678 (5)
C11	0.7569 (4)	1.2516 (4)	0.2460 (3)	0.0391 (13)	0.678 (5)
H11	0.7171	1.3314	0.1952	0.047*	0.678 (5)
C12	0.8665 (4)	1.3237 (4)	0.3151 (3)	0.0433 (17)	0.678 (5)
H12A	0.9237	1.3619	0.2761	0.052*	0.678 (5)
H12B	0.8416	1.4149	0.3504	0.052*	0.678 (5)
C13	0.9280 (3)	1.2023 (4)	0.3896 (3)	0.0424 (16)	0.678 (5)
H13	1.0006	1.2506	0.4358	0.051*	0.678 (5)
C14	0.9677 (3)	1.0649 (5)	0.3362 (3)	0.0434 (16)	0.678 (5)
H14A	1.0238	1.1016	0.2959	0.052*	0.678 (5)
H14B	1.0112	0.9869	0.3855	0.052*	0.678 (5)
C15	0.6689 (3)	1.1999 (5)	0.3060 (3)	0.0374 (13)	0.678 (5)
H15A	0.5951	1.1551	0.2611	0.045*	0.678 (5)
H15B	0.6446	1.2916	0.3413	0.045*	0.678 (5)
C16	0.7294 (3)	1.0775 (4)	0.3804 (3)	0.0303 (12)	0.678 (5)
H16	0.6715	1.0419	0.4204	0.036*	0.678 (5)
C17	0.8409 (4)	1.1462 (5)	0.4491 (2)	0.0377 (13)	0.678 (5)
H17A	0.8175	1.2357	0.4867	0.045*	0.678 (5)
H17B	0.8807	1.0652	0.4977	0.045*	0.678 (5)
C18	0.7658 (3)	0.9387 (3)	0.3261 (3)	0.0261 (11)	0.678 (5)
H18A	0.6934	0.8951	0.2786	0.031*	0.678 (5)
H18B	0.8007	0.8549	0.3745	0.031*	0.678 (5)
C19	1.1068 (3)	0.6541 (5)	0.1049 (3)	0.0280 (7)	
H19A	1.0839	0.6214	0.0339	0.034*	
H19B	1.1599	0.7478	0.1095	0.034*	
C20	1.2026 (3)	0.5548 (4)	0.2700 (2)	0.0264 (7)	
H20A	1.1261	0.5502	0.2926	0.032*	
H20B	1.2384	0.6610	0.2858	0.032*	
C21	1.2901 (3)	0.4310 (4)	0.3246 (3)	0.0271 (7)	
H21A	1.3693	0.4423	0.3067	0.033*	
H21B	1.3037	0.4469	0.3976	0.033*	
C22	1.2169 (3)	0.2464 (4)	0.1899 (2)	0.0283 (7)	
H22A	1.1828	0.1395	0.1733	0.034*	
H22B	1.2933	0.2546	0.1674	0.034*	
C23	1.1266 (3)	0.3703 (4)	0.1372 (2)	0.0241 (6)	
H23A	1.1094	0.3543	0.0640	0.029*	
H23B	1.0493	0.3595	0.1580	0.029*	
C24	1.3055 (3)	0.1486 (4)	0.3583 (2)	0.0220 (6)	
C25	1.3170 (3)	0.1532 (4)	0.4616 (2)	0.0295 (7)	
H25	1.2840	0.2397	0.4902	0.035*	
C26	1.3757 (3)	0.0341 (5)	0.5226 (2)	0.0327 (8)	
H26	1.3825	0.0393	0.5925	0.039*	
C27	1.4254 (3)	−0.0945 (4)	0.4823 (3)	0.0314 (7)	
H27	1.4663	−0.1761	0.5244	0.038*	
C28	1.4139 (3)	−0.1007 (5)	0.3811 (3)	0.0313 (7)	
H28	1.4466	−0.1881	0.3531	0.038*	
C29	1.3552 (3)	0.0193 (4)	0.3184 (2)	0.0267 (6)	
H29	1.3488	0.0134	0.2485	0.032*	
C10'	0.9726 (4)	1.0898 (7)	0.2942 (5)	0.041 (3)	0.322 (5)
H10C	1.0445	1.0199	0.3129	0.050*	0.322 (5)
H10D	0.9787	1.1499	0.2340	0.050*	0.322 (5)
C11'	0.9728 (5)	1.2025 (6)	0.3780 (4)	0.037 (3)	0.322 (5)
H11B	1.0480	1.2687	0.3899	0.045*	0.322 (5)
C12'	0.9704 (5)	1.1144 (8)	0.4720 (4)	0.041 (3)	0.322 (5)
H12C	0.9674	1.1897	0.5263	0.049*	0.322 (5)
H12D	1.0445	1.0492	0.4933	0.049*	0.322 (5)
C13'	0.8595 (5)	1.0112 (6)	0.4507 (3)	0.030 (2)	0.322 (5)
H13'	0.8571	0.9485	0.5116	0.036*	0.322 (5)
C14'	0.8619 (6)	0.9014 (5)	0.3658 (3)	0.034 (3)	0.322 (5)
H14C	0.7912	0.8294	0.3553	0.041*	0.322 (5)
H14D	0.9366	0.8364	0.3832	0.041*	0.322 (5)
C15'	0.8633 (6)	1.3077 (5)	0.3507 (6)	0.037 (4)	0.322 (5)
H15C	0.8626	1.3817	0.4061	0.045*	0.322 (5)
H15D	0.8657	1.3702	0.2905	0.045*	0.322 (5)
C16'	0.7514 (5)	1.2067 (5)	0.3305 (4)	0.027 (2)	0.322 (5)
H16'	0.6780	1.2760	0.3129	0.032*	0.322 (5)
C17'	0.7481 (5)	1.1129 (8)	0.4221 (4)	0.021 (2)	0.322 (5)
H17C	0.6744	1.0459	0.4088	0.025*	0.322 (5)
H17D	0.7448	1.1853	0.4778	0.025*	0.322 (5)
C18'	0.7507 (4)	1.0967 (6)	0.2452 (4)	0.029 (2)	0.322 (5)
H18C	0.6754	1.0329	0.2312	0.035*	0.322 (5)
H18D	0.7521	1.1585	0.1846	0.035*	0.322 (5)

### Atomic displacement parameters (Å^2^)

**Table:**

	*U*^11^	*U*^22^	*U*^33^	*U*^12^	*U*^13^	*U*^23^
S1	0.0281 (4)	0.0282 (4)	0.0246 (3)	−0.0012 (3)	0.0061 (3)	0.0004 (3)
N1	0.0228 (12)	0.0208 (13)	0.0263 (13)	0.0058 (10)	0.0115 (10)	0.0054 (10)
N2	0.0273 (13)	0.0260 (14)	0.0299 (14)	0.0066 (11)	0.0044 (11)	−0.0023 (11)
N3	0.0204 (12)	0.0262 (14)	0.0293 (14)	0.0050 (11)	0.0062 (10)	−0.0020 (11)
N4	0.0222 (12)	0.0271 (15)	0.0279 (13)	0.0022 (11)	0.0077 (10)	0.0019 (11)
N5	0.0290 (13)	0.0241 (13)	0.0196 (12)	0.0017 (11)	0.0050 (10)	−0.0029 (10)
C1	0.0254 (14)	0.0236 (15)	0.0195 (13)	0.0044 (12)	0.0080 (11)	0.0064 (12)
C2	0.0282 (15)	0.0243 (16)	0.0229 (14)	0.0048 (13)	0.0092 (12)	0.0059 (12)
C3	0.0234 (15)	0.0252 (17)	0.0460 (19)	0.0028 (13)	0.0191 (14)	0.0057 (14)
C4	0.043 (2)	0.038 (2)	0.062 (3)	0.0016 (18)	0.0353 (19)	0.0087 (19)
C5	0.050 (3)	0.042 (3)	0.112 (4)	−0.014 (2)	0.061 (3)	−0.008 (3)
C6	0.0262 (19)	0.044 (3)	0.130 (5)	0.0000 (19)	0.035 (3)	−0.008 (3)
C7	0.0249 (18)	0.040 (2)	0.109 (4)	0.0076 (18)	0.008 (2)	0.011 (3)
C8	0.0251 (17)	0.034 (2)	0.063 (3)	0.0058 (15)	0.0117 (16)	0.0174 (18)
C9	0.055 (2)	0.0231 (16)	0.0432 (18)	−0.0005 (17)	0.0344 (17)	0.0007 (16)
C10	0.052 (3)	0.025 (3)	0.050 (3)	0.007 (2)	0.023 (3)	−0.004 (2)
C11	0.060 (3)	0.024 (3)	0.038 (3)	0.007 (2)	0.023 (3)	0.001 (2)
C12	0.063 (4)	0.016 (3)	0.062 (4)	−0.006 (3)	0.038 (3)	−0.004 (3)
C13	0.050 (4)	0.038 (3)	0.048 (3)	0.006 (3)	0.029 (3)	−0.017 (3)
C14	0.040 (3)	0.054 (4)	0.030 (3)	0.011 (3)	−0.004 (2)	−0.019 (3)
C15	0.041 (3)	0.033 (3)	0.041 (3)	0.011 (2)	0.016 (2)	−0.008 (2)
C16	0.033 (3)	0.035 (3)	0.026 (3)	−0.002 (2)	0.014 (2)	−0.004 (2)
C17	0.046 (3)	0.036 (3)	0.033 (3)	−0.003 (3)	0.012 (2)	−0.002 (2)
C18	0.030 (2)	0.026 (2)	0.026 (2)	0.0007 (19)	0.0130 (19)	0.0026 (17)
C19	0.0216 (14)	0.0293 (17)	0.0351 (17)	0.0068 (13)	0.0109 (13)	0.0053 (14)
C20	0.0272 (15)	0.0232 (15)	0.0280 (16)	−0.0008 (12)	0.0052 (12)	−0.0068 (12)
C21	0.0256 (15)	0.0261 (16)	0.0279 (16)	−0.0018 (13)	0.0029 (12)	−0.0019 (12)
C22	0.0374 (17)	0.0249 (16)	0.0215 (15)	0.0071 (14)	0.0050 (13)	−0.0010 (12)
C23	0.0283 (15)	0.0235 (16)	0.0194 (14)	0.0037 (13)	0.0035 (11)	−0.0025 (12)
C24	0.0175 (13)	0.0237 (15)	0.0236 (14)	−0.0036 (12)	0.0028 (11)	−0.0018 (12)
C25	0.0301 (16)	0.0308 (17)	0.0278 (16)	0.0007 (14)	0.0071 (13)	−0.0021 (13)
C26	0.0306 (16)	0.042 (2)	0.0238 (15)	−0.0086 (15)	0.0025 (12)	0.0020 (13)
C27	0.0246 (15)	0.0306 (18)	0.0369 (18)	−0.0032 (14)	0.0036 (13)	0.0109 (14)
C28	0.0275 (16)	0.0262 (17)	0.0398 (19)	0.0012 (14)	0.0075 (14)	0.0020 (14)
C29	0.0238 (14)	0.0274 (17)	0.0294 (15)	−0.0007 (14)	0.0074 (11)	−0.0002 (13)
C10'	0.052 (5)	0.041 (5)	0.031 (5)	0.018 (4)	0.009 (4)	−0.019 (4)
C11'	0.042 (5)	0.040 (5)	0.036 (5)	0.017 (4)	0.020 (4)	0.005 (4)
C12'	0.040 (5)	0.039 (5)	0.047 (5)	0.004 (4)	0.016 (4)	0.007 (4)
C13'	0.030 (4)	0.027 (4)	0.036 (4)	0.001 (4)	0.011 (3)	0.003 (4)
C14'	0.035 (4)	0.028 (4)	0.044 (4)	−0.005 (4)	0.020 (4)	0.000 (4)
C15'	0.042 (5)	0.034 (5)	0.036 (5)	0.003 (4)	0.009 (4)	−0.002 (4)
C16'	0.017 (4)	0.027 (4)	0.033 (4)	0.004 (3)	−0.003 (3)	0.000 (3)
C17'	0.021 (4)	0.022 (4)	0.025 (4)	0.000 (3)	0.015 (3)	0.002 (3)
C18'	0.035 (4)	0.032 (4)	0.022 (4)	−0.006 (3)	0.011 (3)	0.001 (3)

### Geometric parameters (Å, °)

**Table:**

S1—C1	1.673 (3)	C17—H17A	0.9900
N1—C1	1.381 (4)	C17—H17B	0.9900
N1—C2	1.390 (4)	C18—H18A	0.9900
N1—C3	1.439 (4)	C18—H18B	0.9900
N2—C2	1.301 (4)	C19—H19A	0.9900
N2—N3	1.387 (4)	C19—H19B	0.9900
N3—C1	1.347 (4)	C20—C21	1.514 (5)
N3—C19	1.478 (4)	C20—H20A	0.9900
N4—C19	1.447 (4)	C20—H20B	0.9900
N4—C20	1.462 (4)	C21—H21A	0.9900
N4—C23	1.460 (4)	C21—H21B	0.9900
N5—C24	1.418 (4)	C22—C23	1.525 (4)
N5—C21	1.468 (4)	C22—H22A	0.9900
N5—C22	1.468 (4)	C22—H22B	0.9900
C2—C9	1.546 (4)	C23—H23A	0.9900
C3—C4	1.382 (5)	C23—H23B	0.9900
C3—C8	1.378 (5)	C24—C25	1.400 (4)
C4—C5	1.403 (6)	C24—C29	1.403 (5)
C4—H4	0.9500	C25—C26	1.378 (5)
C5—C6	1.363 (8)	C25—H25	0.9500
C5—H5	0.9500	C26—C27	1.401 (5)
C6—C7	1.375 (7)	C26—H26	0.9500
C6—H6	0.9500	C27—C28	1.372 (5)
C7—C8	1.396 (5)	C27—H27	0.9500
C7—H7	0.9500	C28—C29	1.396 (5)
C8—H8	0.9500	C28—H28	0.9500
C9—C14	1.491 (2)	C29—H29	0.9500
C9—C18'	1.492 (2)	C10'—C11'	1.499 (2)
C9—C18	1.5111 (19)	C10'—H10C	0.9900
C9—C10'	1.512 (2)	C10'—H10D	0.9900
C9—C14'	1.512 (2)	C11'—C15'	1.501 (2)
C9—C10	1.518 (2)	C11'—C12'	1.501 (2)
C10—C11	1.503 (2)	C11'—H11B	1.0000
C10—H10A	0.9900	C12'—C13'	1.502 (2)
C10—H10B	0.9900	C12'—H12C	0.9900
C11—C12	1.502 (2)	C12'—H12D	0.9900
C11—C15	1.504 (2)	C13'—C14'	1.501 (2)
C11—H11	1.0000	C13'—C17'	1.501 (2)
C12—C13	1.502 (2)	C13'—H13'	1.0000
C12—H12A	0.9900	C14'—H14C	0.9900
C12—H12B	0.9900	C14'—H14D	0.9900
C13—C17	1.503 (2)	C15'—C16'	1.500 (2)
C13—C14	1.503 (2)	C15'—H15C	0.9900
C13—H13	1.0000	C15'—H15D	0.9900
C14—H14A	0.9900	C16'—C18'	1.499 (2)
C14—H14B	0.9900	C16'—C17'	1.501 (2)
C15—C16	1.504 (2)	C16'—H16'	1.0000
C15—H15A	0.9900	C17'—H17C	0.9900
C15—H15B	0.9900	C17'—H17D	0.9900
C16—C17	1.502 (2)	C18'—H18C	0.9900
C16—C18	1.505 (2)	C18'—H18D	0.9900
C16—H16	1.0000		
			
C1—N1—C2	108.1 (2)	H18A—C18—H18B	108.2
C1—N1—C3	121.7 (3)	N4—C19—N3	116.3 (3)
C2—N1—C3	130.0 (3)	N4—C19—H19A	108.2
C2—N2—N3	105.4 (3)	N3—C19—H19A	108.2
C1—N3—N2	112.3 (2)	N4—C19—H19B	108.2
C1—N3—C19	129.8 (3)	N3—C19—H19B	108.2
N2—N3—C19	117.6 (3)	H19A—C19—H19B	107.4
C19—N4—C20	113.0 (3)	N4—C20—C21	110.2 (3)
C19—N4—C23	114.5 (3)	N4—C20—H20A	109.6
C20—N4—C23	110.8 (2)	C21—C20—H20A	109.6
C24—N5—C21	115.1 (2)	N4—C20—H20B	109.6
C24—N5—C22	115.6 (3)	C21—C20—H20B	109.6
C21—N5—C22	110.9 (3)	H20A—C20—H20B	108.1
N3—C1—N1	104.0 (3)	N5—C21—C20	110.6 (3)
N3—C1—S1	128.7 (2)	N5—C21—H21A	109.5
N1—C1—S1	127.3 (2)	C20—C21—H21A	109.5
N2—C2—N1	110.2 (3)	N5—C21—H21B	109.5
N2—C2—C9	122.7 (3)	C20—C21—H21B	109.5
N1—C2—C9	127.1 (3)	H21A—C21—H21B	108.1
C4—C3—C8	121.3 (3)	N5—C22—C23	109.1 (3)
C4—C3—N1	119.5 (3)	N5—C22—H22A	109.9
C8—C3—N1	119.2 (3)	C23—C22—H22A	109.9
C3—C4—C5	118.4 (4)	N5—C22—H22B	109.9
C3—C4—H4	120.8	C23—C22—H22B	109.9
C5—C4—H4	120.8	H22A—C22—H22B	108.3
C6—C5—C4	120.7 (4)	N4—C23—C22	109.8 (3)
C6—C5—H5	119.7	N4—C23—H23A	109.7
C4—C5—H5	119.7	C22—C23—H23A	109.7
C5—C6—C7	120.4 (4)	N4—C23—H23B	109.7
C5—C6—H6	119.8	C22—C23—H23B	109.7
C7—C6—H6	119.8	H23A—C23—H23B	108.2
C6—C7—C8	120.2 (5)	C25—C24—C29	118.2 (3)
C6—C7—H7	119.9	C25—C24—N5	118.9 (3)
C8—C7—H7	119.9	C29—C24—N5	122.9 (3)
C3—C8—C7	119.0 (4)	C26—C25—C24	120.9 (3)
C3—C8—H8	120.5	C26—C25—H25	119.5
C7—C8—H8	120.5	C24—C25—H25	119.5
C14—C9—C18'	113.4 (3)	C25—C26—C27	120.6 (3)
C14—C9—C18	112.7 (2)	C25—C26—H26	119.7
C18'—C9—C10'	108.8 (3)	C27—C26—H26	119.7
C18'—C9—C14'	110.4 (3)	C28—C27—C26	119.0 (3)
C10'—C9—C14'	104.2 (3)	C28—C27—H27	120.5
C14—C9—C10	108.7 (3)	C26—C27—H27	120.5
C18—C9—C10	105.9 (2)	C27—C28—C29	121.1 (3)
C14—C9—C2	112.6 (2)	C27—C28—H28	119.4
C18'—C9—C2	127.6 (3)	C29—C28—H28	119.4
C18—C9—C2	112.3 (2)	C28—C29—C24	120.2 (3)
C10'—C9—C2	102.9 (3)	C28—C29—H29	119.9
C14'—C9—C2	100.4 (3)	C24—C29—H29	119.9
C10—C9—C2	103.8 (2)	C11'—C10'—C9	111.7 (3)
C11—C10—C9	110.5 (2)	C11'—C10'—H10C	109.3
C11—C10—H10A	109.5	C9—C10'—H10C	109.3
C9—C10—H10A	109.5	C11'—C10'—H10D	109.3
C11—C10—H10B	109.5	C9—C10'—H10D	109.3
C9—C10—H10B	109.5	H10C—C10'—H10D	107.9
H10A—C10—H10B	108.1	C10'—C11'—C15'	109.6 (3)
C12—C11—C10	109.5 (3)	C10'—C11'—C12'	110.6 (3)
C12—C11—C15	109.1 (3)	C15'—C11'—C12'	109.1 (3)
C10—C11—C15	109.6 (3)	C10'—C11'—H11B	109.2
C12—C11—H11	109.5	C15'—C11'—H11B	109.2
C10—C11—H11	109.5	C12'—C11'—H11B	109.2
C15—C11—H11	109.5	C11'—C12'—C13'	108.0 (3)
C13—C12—C11	109.4 (2)	C11'—C12'—H12C	110.1
C13—C12—H12A	109.8	C13'—C12'—H12C	110.1
C11—C12—H12A	109.8	C11'—C12'—H12D	110.1
C13—C12—H12B	109.8	C13'—C12'—H12D	110.1
C11—C12—H12B	109.8	H12C—C12'—H12D	108.4
H12A—C12—H12B	108.2	C14'—C13'—C17'	108.8 (3)
C12—C13—C17	109.4 (3)	C14'—C13'—C12'	110.0 (3)
C12—C13—C14	109.9 (3)	C17'—C13'—C12'	109.2 (3)
C17—C13—C14	109.6 (3)	C14'—C13'—H13'	109.6
C12—C13—H13	109.3	C17'—C13'—H13'	109.6
C17—C13—H13	109.3	C12'—C13'—H13'	109.6
C14—C13—H13	109.3	C13'—C14'—C9	111.8 (3)
C9—C14—C13	109.1 (2)	C13'—C14'—H14C	109.3
C9—C14—H14A	109.9	C9—C14'—H14C	109.3
C13—C14—H14A	109.9	C13'—C14'—H14D	109.3
C9—C14—H14B	109.9	C9—C14'—H14D	109.3
C13—C14—H14B	109.9	H14C—C14'—H14D	107.9
H14A—C14—H14B	108.3	C16'—C15'—C11'	108.6 (3)
C11—C15—C16	108.8 (2)	C16'—C15'—H15C	110.0
C11—C15—H15A	109.9	C11'—C15'—H15C	110.0
C16—C15—H15A	109.9	C16'—C15'—H15D	110.0
C11—C15—H15B	109.9	C11'—C15'—H15D	110.0
C16—C15—H15B	109.9	H15C—C15'—H15D	108.3
H15A—C15—H15B	108.3	C18'—C16'—C15'	110.2 (3)
C17—C16—C15	109.9 (3)	C18'—C16'—C17'	109.5 (3)
C17—C16—C18	109.1 (3)	C15'—C16'—C17'	109.7 (3)
C15—C16—C18	109.5 (2)	C18'—C16'—H16'	109.1
C17—C16—H16	109.4	C15'—C16'—H16'	109.1
C15—C16—H16	109.4	C17'—C16'—H16'	109.1
C18—C16—H16	109.4	C16'—C17'—C13'	109.6 (3)
C13—C17—C16	110.0 (2)	C16'—C17'—H17C	109.7
C13—C17—H17A	109.7	C13'—C17'—H17C	109.7
C16—C17—H17A	109.7	C16'—C17'—H17D	109.7
C13—C17—H17B	109.7	C13'—C17'—H17D	109.7
C16—C17—H17B	109.7	H17C—C17'—H17D	108.2
H17A—C17—H17B	108.2	C9—C18'—C16'	110.3 (2)
C16—C18—C9	109.6 (2)	C9—C18'—H18C	109.6
C16—C18—H18A	109.7	C16'—C18'—H18C	109.6
C9—C18—H18A	109.7	C9—C18'—H18D	109.6
C16—C18—H18B	109.7	C16'—C18'—H18D	109.6
C9—C18—H18B	109.7	H18C—C18'—H18D	108.1
			
C2—N2—N3—C1	0.1 (3)	C18—C16—C17—C13	61.0 (3)
C2—N2—N3—C19	173.9 (3)	C17—C16—C18—C9	−57.2 (3)
N2—N3—C1—N1	0.1 (3)	C15—C16—C18—C9	63.1 (3)
C19—N3—C1—N1	−172.7 (3)	C14—C9—C18—C16	56.4 (3)
N2—N3—C1—S1	179.6 (2)	C10—C9—C18—C16	−62.4 (3)
C19—N3—C1—S1	6.8 (5)	C2—C9—C18—C16	−175.1 (3)
C2—N1—C1—N3	−0.2 (3)	C20—N4—C19—N3	−50.8 (4)
C3—N1—C1—N3	174.9 (3)	C23—N4—C19—N3	77.3 (4)
C2—N1—C1—S1	−179.7 (2)	C1—N3—C19—N4	−91.6 (4)
C3—N1—C1—S1	−4.6 (4)	N2—N3—C19—N4	95.9 (3)
N3—N2—C2—N1	−0.2 (3)	C19—N4—C20—C21	−172.1 (3)
N3—N2—C2—C9	179.8 (3)	C23—N4—C20—C21	57.8 (3)
C1—N1—C2—N2	0.2 (3)	C24—N5—C21—C20	−169.1 (3)
C3—N1—C2—N2	−174.3 (3)	C22—N5—C21—C20	57.3 (3)
C1—N1—C2—C9	−179.8 (3)	N4—C20—C21—N5	−56.3 (4)
C3—N1—C2—C9	5.7 (5)	C24—N5—C22—C23	168.3 (3)
C1—N1—C3—C4	91.4 (4)	C21—N5—C22—C23	−58.3 (3)
C2—N1—C3—C4	−94.7 (4)	C19—N4—C23—C22	171.3 (3)
C1—N1—C3—C8	−88.0 (4)	C20—N4—C23—C22	−59.5 (3)
C2—N1—C3—C8	85.9 (5)	N5—C22—C23—N4	59.2 (3)
C8—C3—C4—C5	−0.7 (6)	C21—N5—C24—C25	57.9 (4)
N1—C3—C4—C5	179.9 (4)	C22—N5—C24—C25	−170.7 (3)
C3—C4—C5—C6	0.0 (7)	C21—N5—C24—C29	−123.8 (3)
C4—C5—C6—C7	0.2 (8)	C22—N5—C24—C29	7.5 (4)
C5—C6—C7—C8	0.3 (8)	C29—C24—C25—C26	0.1 (5)
C4—C3—C8—C7	1.2 (7)	N5—C24—C25—C26	178.4 (3)
N1—C3—C8—C7	−179.4 (4)	C24—C25—C26—C27	0.1 (5)
C6—C7—C8—C3	−1.0 (7)	C25—C26—C27—C28	−0.5 (5)
N2—C2—C9—C14	−9.7 (4)	C26—C27—C28—C29	0.7 (5)
N1—C2—C9—C14	170.3 (3)	C27—C28—C29—C24	−0.6 (5)
N2—C2—C9—C18'	140.3 (4)	C25—C24—C29—C28	0.1 (4)
N1—C2—C9—C18'	−39.7 (5)	N5—C24—C29—C28	−178.1 (3)
N2—C2—C9—C18	−138.3 (3)	C18'—C9—C10'—C11'	57.1 (4)
N1—C2—C9—C18	41.8 (4)	C14'—C9—C10'—C11'	−60.7 (4)
N2—C2—C9—C10'	13.7 (4)	C2—C9—C10'—C11'	−165.1 (4)
N1—C2—C9—C10'	−166.2 (4)	C9—C10'—C11'—C15'	−58.3 (4)
N2—C2—C9—C14'	−93.6 (4)	C9—C10'—C11'—C12'	62.0 (4)
N1—C2—C9—C14'	86.5 (4)	C10'—C11'—C12'—C13'	−57.6 (4)
N2—C2—C9—C10	107.7 (3)	C15'—C11'—C12'—C13'	63.1 (4)
N1—C2—C9—C10	−72.2 (4)	C11'—C12'—C13'—C14'	57.6 (4)
C14—C9—C10—C11	−59.7 (3)	C11'—C12'—C13'—C17'	−61.8 (4)
C18—C9—C10—C11	61.7 (3)	C17'—C13'—C14'—C9	57.0 (4)
C2—C9—C10—C11	−179.8 (3)	C12'—C13'—C14'—C9	−62.6 (4)
C9—C10—C11—C12	58.5 (3)	C14—C9—C14'—C13'	56.1 (4)
C9—C10—C11—C15	−61.1 (3)	C18'—C9—C14'—C13'	−55.5 (4)
C10—C11—C12—C13	−58.4 (4)	C10'—C9—C14'—C13'	61.2 (4)
C15—C11—C12—C13	61.6 (3)	C2—C9—C14'—C13'	167.5 (4)
C11—C12—C13—C17	−60.0 (3)	C10'—C11'—C15'—C16'	59.0 (4)
C11—C12—C13—C14	60.3 (4)	C12'—C11'—C15'—C16'	−62.2 (4)
C18—C9—C14—C13	−56.6 (4)	C11'—C15'—C16'—C18'	−60.8 (4)
C10—C9—C14—C13	60.5 (3)	C11'—C15'—C16'—C17'	59.9 (4)
C2—C9—C14—C13	175.1 (3)	C18'—C16'—C17'—C13'	61.9 (4)
C12—C13—C14—C9	−61.8 (4)	C15'—C16'—C17'—C13'	−59.2 (4)
C17—C13—C14—C9	58.4 (4)	C14'—C13'—C17'—C16'	−60.0 (4)
C12—C11—C15—C16	−61.3 (3)	C12'—C13'—C17'—C16'	60.1 (4)
C10—C11—C15—C16	58.6 (3)	C10'—C9—C18'—C16'	−57.6 (4)
C11—C15—C16—C17	60.1 (3)	C14'—C9—C18'—C16'	56.1 (4)
C11—C15—C16—C18	−59.7 (3)	C2—C9—C18'—C16'	178.2 (3)
C12—C13—C17—C16	58.7 (3)	C15'—C16'—C18'—C9	61.0 (4)
C14—C13—C17—C16	−61.9 (3)	C17'—C16'—C18'—C9	−59.8 (4)
C15—C16—C17—C13	−59.0 (3)		

### Hydrogen-bond geometry (Å, °)

**Table:**

*D*—H···*A*	*D*—H	H···*A*	*D*···*A*	*D*—H···*A*
C19—H19B···S1^i^	0.99	2.84	3.593 (4)	133

Symmetry codes: (i) −*x*+2, *y*+1/2, −*z*.
